# A General Treatment of Irregularities

**Published:** 1882-02

**Authors:** Walter H. Coffin

**Affiliations:** London


### THE DENTAL REGISTER.

VOL. XXXVI.] **FEBRUARY, 1882** [No. 2.

### A GENERALIZED TREATMENT OF IRREGULARITIES.

*A Paper Read before Section XII. of the International Medical Congress, London, August* 1881.

BY WALTER H. COFFIN, F. C. S.,
LONDON.

The possibilities of regulating are known; but on any case of irregularity presenting for treatment, the practical question will be; What is the best to do, and how is the best to do it? A classification and analysis, in even their infinite variety, of a sufficient number of instances, and the results of their treatment by every possible means, should afford at least an approximate answer to these, and the no less important questions,---what not to do, and how to avoid doing it?

The few models shown have been selected, as fairly representative, from a collection of several thousand, recording the attempted treatment of perhaps a larger proportion than usual of the ordinary irregularities met with in an average practice, and, ---as extending over more than twenty-five years---illustrating the evolution, within certain limits, of an almost generalized method.

A peculiar system thus developed, in use for many years, is no longer a novelty; but having been imperfectly made known, (for which perhaps its advocates are responsible,) and a misconception existing as to its real objects,---those in whose hands it has been an invaluable aid, feel that some account of what they conceive to be its capabilities and limitations may not be unwelcome.

Classifying any large series most conveniently by the mechanical exigencies of each case, in certain of them extraction of possibly sound teeth may, of course, be necessary,---though these are less numerous than usually imagined ;---a possible alternative in many instances affording, (as shown by casts submitted), satisfactory results.

A large class, uncomplicated by crowding, in which aberrant teeth are easily replaced, admit of direct and immediate correction by suitable means (a simplification of which will be alluded to).

Of the remainder, the majority are cases, of every variety, in which the teeth,---not really too large or numerous for the jaw they might symmetrically occupy,---are by some chance of their eruption, irregularly disposed, interlocked, and crowded. Of these it may be affirmed, quite generally, that rectification necessitates the movement of many teeth or all, and an altered shape or outline of the dental arch, for any attempted direct adjustment of individual teeth will be accompanied by such a disturbance more or less extensive. These present the greatest difficulty in regulating by the usual way, especially with a rigid plate; but in the most intricate or the simplest of them, the permissive control of the general tendency of movement during regulation, reduces their successful treatment to comparative ease and certainty. This mechanical *anticipation* of favorable conditions may be illustrated by assuming an incisor to be moved in a crowded arch by any means applied by a plate rigidly embracing the bicuspids and canines, when a certain force in a certain time may complete the operation; but were the plate either abolished, or its symmetrical halves partly independent and free to move relatively in the plane of the arch, less time and force would suffice; and, furthermore, if its halves tend but slightly to seperate by an elastic spring reaction, many cases will require very much less time and force to be exerted on the tooth. The action thus stated in its simplest form is obvious from *a priori* considerations, but was observed, it is believed for the first time, by a singular accident.

Soon after the introduction of vulcanite my father was employing a plate of that material to move an incisor by the swelling of wood. Successive increments of force were resisted until not only was it suddenly in position, but other front teeth were found slightly separated where previously in overlapping contact; the wood, (being nearly on the median line), by lateral expansion, having split the plate down the center. In this instance, as will often be the case, previous "expansion of the arch" by the means usually applied, was certainly not indicated, and therefore not resorted to, although just the slight amount of spreading required was prevented by the rigid construction of the plate. A conviction of this led to a particular method of treating various irregularities, which, as anticipating changes common to them---usually expansive,---has been called somewhat indefinitely an "expansion treatment;" and whose adoption has been abundantly justified by experience.

The troublesome and delicate operation of "expanding the arch," as usually performed, if attempted by the ordinary "jack screw" direct, must be accomplished before other regulating action can generally be commenced, and may then prove to be either excessive or unnecessary. The screw is applicable with care to severe contraction, though inferior to other means; but undivided plates---however thin and elastic,---or hinged plates---however actuated---have not the freedom of movement and adjustability desirable; and the screw is entirely unsuitable for a split plate.

The little device my father calls an "expansion plate," whether used for direct expansion or not, is intrinsically of extreme simplicity, while of complex regulating action; comprising a means--easily embodied in any plate—of conveniently permitting or assisting (instead of hindering or preventing) during regulation, the inevitable changes of the arch naturally accompanying it; and supplementing ordinary expedients with an expansive characteristic. Its distinguishing function depends on the principle of permitting a relative motion, or maintaining a particular controllable reaction, between two semi-independent parts, usually its symmetrical halves.

If required, any force however small, is sufficient, if exerted continuously over a certain distance, with not too rapidly diminishing intensity. Mere repulsion, however, between two points on a split plate, is an unstable system, and uncontrollable. Allowing a certain freedom of motion, means must be provided for restraining it, and maintaining by a yielding guidance any desired degree of parallelism.

Difficulties attended the first realization of these conditions; but it is found that a wire spring of certain form, if a constructive part of the plate, will itself meet all requirements.

Modifications of the arrangement found most convenient and satisfactory are exhibited;—after actual use, and in different stages of construction.

The general form* may be described as a rather thin vulcanite plate, capping and clasping some or all of the biscupids and molars, and fitting the lingual surfaces of anterior teeth; but divided along the median line into two distinct halves, connected, however, by a slight steel wire, so disposed that, while guiding and limiting their relative motion, its tension exerted between them may be perfectly determined and varied in direction and magnitude.

When necessary in such a plate to establish the spring reaction, a surprisingly small, almost imperceptible *stress*, even so distributed, and against a widespread resistance, if continuously maintained and suitably applied, suffices to produce any degree of motion desired.

The perfection of the model must be insisted upon; as an *entire* plate may fit well and securely, and yet both its halves so loose when *divided* as to be useless; while, on the other hand, the halves of a split plate may be easily fitted, which before division could not possibly be inserted. The best impressions have been obtained with the preparations of gutta-percha or ballata gum, no other material affording with ease the absolute fit essential for a split plate. Their physical property (when in good condition and at the right temperature,) of being elastic and recoverable to rapid changes, reproducing, if inserted slowly and

* See Coleman's *Dent. Surg.* 1881, pp. 63-64.

removed quickly, the most intricate undercuts just sufficiently,---and affording by the slight contraction in cooling just enough shrinkage,---for a thin hard rubber copy to fit tightly. A delicate and elastic vulcanite plate from a good gutta-percha impression,---if the model be vulcanized upon direct, and not touched to accentuate undercuts or correct imperfections,---will generally spring over the teeth with so absolute a fit that its removal may even be embarrassing; but until divided its insertion is not usually attempted.

Trials of the metals and their alloys proved the superiority for springs of apparently so undesirable a material as steel.

The almost insuperable difficulty of satisfactorily tempering bent soft steel without deformation of shape, was obviated by the use of pianoforte wire; as possessing very uniform texture, temper permitting it to be fashioned and used without heating, and a surface hardness and burnish which greatly tend to its preservation. To coat this wire with other substances was found unnecessary and undesirable. The behavior of steel to the fluids of the mouth is such that, if hard and bright at first, and continuously immersed in average saliva, it generally assumes a black polished surface, the smooth fairly adherent tarnish being apparently insoluble. A diameter of between three and four-hundredths of an inch, (about 0.035 inch), is most suitable, as of this a convenient length of from 1 to 2½ inches exerts an appropriate tension in average cases. The force--varying inversely as the length---may be thus determined within those limits, beyond which a different size is required.

The extremities being buried rigidly in the vulcanite, the uncovered and active portion of the wire, emerging from selected points in the alveolar region, should be entirely on the lingual side of the plate, nicely fitting, but free to move upon its surface. The wire between its attachments may be in a simple curve, when, for localized action (as exclusively posterior expansion), it will urge a relative motion or rotation about some point; but that every kind of motion may be established, there must be one or more raversals of curvature, by either a single couple of opposite curves, or any number of alternately contrary ones,

(preferably *odd,*) approximately balanced, and as large and symmetrical as possible. Small bends or angles being avoided, the disposition of the wire may otherwise entirely conform to its most convenient adaptation to the particular plate. A serviceable form for an upper general expander is a three or five-curve serpentine figure, like a rounded capital W.

The spring being shaped to fit as nearly as may be the palatal surface of an upper model, or the lingual surface of a lower, has its ends for half an inch (without being softened) slightly flattened and roughened, and so bent towards the model as to raise it uniformly from the surface to a distance of about the desired thickness of the plate, and the portion to be inserted in the rubber tinned or coated with common solder. To several points upon its exposed part, short ends, or loops of binding wire, are twisted to better secure it in the plaster investment. When in shape, it must be free from tension, and attached in the vulcanization to the plate, which is made *entire* and afterwards divided.

The plate being modelled in wax, the spring is placed on the surface, with its ends buried within, and when removed by the counterpart, protected from the rubber by tin foil before packing. Finished in the usual manner, entire, the plate is divided with a fine saw, the edges and corners of the cleft being well rounded and smoothed. This, with care, may be done without imparting tension or twist to the spring, which is important.

The plate should be inserted and worn in the mouth without tension for a day or two, to first eliminate causes of irritation not due to its expansive action, and sooner induce toleration of its presence. Any expansive force required may then be established, and its right direction secured, by stretching the two halves with a slight exaggeration into the relative positions towards which it is desired they should tend to move. This however, may require correction and modification by observing its effect in the mouth. The amount of stress, which is not so conveniently diminished as increased, should be small at first, especially in plates whose expansive function may be simply permissive or auxiliary to other regulating action. It may safely be entrusted to the patient (even quite young) to frequently remove, clean, and replace, after duly cautioning, without fear of disturbing its adjustment.

The experience gained of steel wire has led to its almost exclusive adoption for ordinary regulating purposes, as spring levers acting directly on the teeth, for pulling, pushing, or rotating; and, being permanently fixed to the plate, their convenience, adjustability, and many adaptations, are remarkable. Combined with a split plate they are found to replace with advantage, screws, inclined planes, wedges, levers, and ligatures, in many of their local uses; and, moreover, are practicable where nothing else can be applied. In fact, by the gradual simplification of means, and the elimination of uncertain devices, a delicate split plate, with one or two short lengths of small wire closely fitting its surface, is often now to those who employ it, the representative of the wondrous combinations of nearly all the known "mechanical powers" that once exercised their ingenuity. Where in simple cases a plate is unnecessary, with an inch or two of steel wire and elastic rubber tube, efficient expedients may frequently be improvised.

When direct expansion of the arch is indicated, the thin spring reaction plate, fitting closely the palate, teeth, and tissues covering the alveoli, and not filling the mouth or impeding its functions, will, with the minimum of trouble and attention, effect any desired degree of that spreading and moulding of the mouth which it is well known may, to a surprising extent, be rapidly and almost painlessly produced. So easily is this done that caution must be observed not to inadvertently exceed the intended results, and the temptation to so readily make room for crowded-out teeth, though often with gratifying effect, must sometimes be resisted. Indeed, the extreme facility and rapidity of general expansion by the split plate, has in the hands of some without experience of its limitations and abuses, been to the prejudice of the method.

Among the uses of direct expansion, its interesting application to the operative treatment of caries, and to slightly overlapping incisors, may be discussed together.

In young mouths where interstitial decay of incisors closely in contact almost defies successful treatment, any operation attempted, whether of separating surfaces, shaping or filling cavities, or restoring contours, will, of course, be facilitated by gaining ever so small a space between them. Paradoxical as it may seem, it is actually less painful and troublesome to secure ample spaces between all the front teeth at once, than to wedge two of them apart in the ordinary way; with the advantage of easily maintaining their separation without irritation during any course of treatment.

A plate exerting anterior expansion will rapidly---and usually almost painlessly---separate the incisors, and by a suitable adjustment of the bearing surfaces, may readily be caused to distribute spaces equally between all the front teeth. Their slight tenderness soon disappears if held by the same or another plate, and they may be kept apart without fear of their not coming together again. In nerve inflammation, caution will suggest itself; but an accident has not occurred from that cause. If the incisors are just overlapping, slight expansion, with suitable disposition of the bearing surface afterwards, usually permits the natural action of the lips to straighten them.

In some of these cases it may be necessary to partly maintain the expansion of posterior teeth by a suitable retaining plate, exposing their articulating surfaces till the bite is readjusted by those of the other jaw accommodating themselves. Where expansion in one jaw is considerable, the bite may require a slight mechanical expansion of the other, which, for this purpose, and assisted by the articulation itself, is easily attained.

Expansion, where posterior teeth wrongly articulate, presents little difficulty when the conditions are symmetrical on both sides; when otherwise, or confined to one side only, a differential or even a unilateral effect may be produced, according to the number and kind of teeth the action of the plate is distributed or concentrated upon, and the arrangement of its articulating surfaces. In differential expansion (which may occur unexpectedly) the relative motions appear disproportional, that is, seem to vary (inversely) *more* than the estimated resistances.

Lastly, the obvious application of the divided spring plate to narrow mis-shaped mouths with high contracted roofs, unfortunately also presents considerable difficulty. These cases admit of very great improvement, (as illustrated by models exhibited,) where circumstances are favorable for expansion: as when the bite can be either kept normal or made so, and the jaw is not really so contracted as the dental arch makes it appear; for such the split reaction plate is peculiarly adapted and unsurpassable, if used with caution.

In several cases accompanied by prominent incisors, after only slight posterior expansion, considerable spontaneous recession of the incisors has occurred, probably by the action of the lips, before special mechanical means were employed.

In the models of many cases treated, will be observed the profound alteration in shape the palate vault appears to have undergone. Inflammatory symptoms observed along the median line, corresponding to anatomical relations, (possibly caused by the edges of the cleft plate,) have suggested certain bone disturbances; but though extensive changes seem otherwise almost inexplicable, inter-osseous action can only be conjectured.

While expansion is most readily performed in young mouths, especially under 16, the only limitations of age would seem to be the diminished power of restoring fixity after movement, which must be inferred of advanced age; though in a case of considerable expansion at 45, the teeth became perfectly firm afterwards.

It is hardly necessary to dwell on the desirability of the greatest possible simplicity of regulating devices, in principle and details; and the self-maintaining adjustability of plates, that they may be as independent as possible of any control by the patient, whose co-operation should be confined to their perfect cleanliness only, and the health and comfort of the mouth. But it may, perhaps, be pointed out, that greater beauty and finish of plates than is customary is not without effect in assuring care and attention to their good condition.

Great credit is due for working out certain details of expansion plates, to Mr. Peter Headridge, of Manchester, for many years assistant to my father. This gentleman even obtained a patent for some constructive particulars, which, however, he very advisedly abandoned. The curious are referred to specification 1101, 1869.

In final justification, the advocates of a method they find to simplify and trust may extend the treatment of irregularities, appeal to their record of results, which—of whatever real importance or value—would have been difficult or impossible to otherwise attain; and have ventured at such length to detail their procedure, for confirmation or criticism by others.

---

In discussion upon the paper,

Dr. ROSENTHAL (*Liege*) having employed Dr. Coffin's system for eight years with great satisfaction, and steadily increasing success, spoke in praise of the simplicity of Dr. Coffin's regulating plates, as affording an easy, rapid, and safe method of treatment. He had been surprised and gratified with results he thought impossible, and described an extreme case, in which the patient, whose speech was at first unintelligible, had ultimately a good speaking and singing voice.

Dr. CUNNINGHAM (*Wisbech*) confessed to the great assistance he had derived from Dr. Coffin's expansion plates, the construction and use of which, however, depended so greatly on details, that he attributed his own success largely to the personal instruction and advice of Dr. Coffin. He, therefore, regretted that the paper omitted many important points, as, for instance the description of the use of gutta-percha, which (though many may think they know all about) he found by Dr. Coffin's peculiar method to revolutionize the taking of impressions. He thought that all who really tried this method of expansion—which deserved wider recognition—would find, as he had found, many unexpected and useful applications of the split plate.

Dr. DENTZ (*Utrecht*) hoped that the paper might be printed at once, for distribution before its official and long-delayed publication in the "*Transactions*" of the Congress.

Mr. SPENCE BATE, F. R. S., as Vice President, thought there would be no difficulty about that.

Dr. Gunning (*New York*), through the Secretary, Mr. Charles Tomes, F. R. S., advocated regulation of teeth at a much earlier age than is usually the custom.

Mr. Oakley Coles, on the "*Origin* and *Treatment* of *certain forms of Irregularity*" admitted that not only the dental arch, but in many cases the jaw itself must be expanded and the teeth regulated afterwards.

Dr. Iszlai Jozsef (*Budapest*) spoke on "*Prognathism*" its measurement, classification, and nomenclature.

The Chairman, remarking on the very practical nature of the Paper and demonstrations illustrating Dr. Coffin's system, and the interesting debate on the subject, called on

The Author, who, in reply, thanking those who so kindly added valuable testimony of experience most gratifying to him, regretted his inability within the limits of time to satisfactorily treat many technical points merely referred to, or omitted altogether; and begged those who had honored him with their presence and attention, to further favor him with enquiries or criticism.

The paper was illustrated by more than 500 old regulating plates which had been actually used; about 400 being "expansion plates," upper and lower, symmetrically and unsymmetrically divided, of which nearly 200 were "simple expanders," some 200 embodying other regulating devices with "expansion;" the remainder showing the application of pianoforte wire in ordinary plates for general regulating purposes.

There were also specimens, with demonstrations, shewing, at different stages, details of their mode of construction.

The models exhibited in the Museum of the Congress, at Burlington House, of forty typical cases, (recording by three or more casts to each, the conditions before, during, and after treatment), were classified as illustrating—

1. Expansion auxiliary to ordinary regulating.
    (*a*) In simple crowding.
    (*b*) For rotation and alignment.

2. General expansion.
    (*a*) For operative treatment of caries.
    (*b*) For mis-articulation.
    (*c*) *Versus* extraction of misplaced teeth.
    (*d*) For prominent incisors.
    (*e*) For contracted, narrow, or mis-shaped arch.
3. Applications of steel wire to every kind of ordinary regulating.
    (*a*) Alone, without plate or accessories, for alignment or rotation.
    (*b*) Combined with elastic ligatures.
    (*c*) With an ordinary plate for moving, shortening, lengthening, and rotating teeth.
4. Combinations of the above.

